# Monthly Summary

**Published:** 1897-02

**Authors:** 


### Monthly Summary.

A CASE OF PYORRHEA ALVEOLARIS.—I desire to relate a case where the patient was salivated some twenty years ago, and his teeth since have frequently troubled him more or less, not with any discharge from the gum, but occasionally a certain tooth would be attacked with calcic abscess. Through these abscesses he has lost three or four teeth. I have had him under my care for about eight years, and for the last four or five years his teeth have been comparatively comfortable. But this summer he went down to French Lick Springs and stayed there ten days. He drank the water, and that apparently brought back his old trouble; that is, all of the symptoms of salivation returned. The gums became inflamed, the peridental membranes very sensitive, and the gums bled profusely in the morning on rising. His teeth were excessively sore in the morning. I gave him a sitting last week and searched for calculus, but did not find very much. I gave him a treatment, using iodide of zinc. He reported yesterday that his gums were in very much better condition. There has been no bleeding since the treatment. The soreness is gradually subsiding and he is feeling quite comfortable.

The question I desire to raise is this, What was there about the water, if it was the water, that brought back the old trouble? The trouble is apparently easily controlled, but it was evidently a recurrence of the old difficulty.—Dr. C. N. Johnson, "Western Dental Journal."

---

METAL DIES DIRECT FROM IMPRESSION.—Dr. E. I. Woodbury, *Dental Cosmos*, has a method of making dies in metal direct from the impression. The material for the impression is fine clay or a clay compound, with an equal part of plaster which is the aluminous compound he uses. It will not shrink or expand, and is also a good investment for soldering. He uses a perforated tray, made in parts composed of an alloy of two per cent. copper with aluminum. This will stand the heat of the temperature at which the metal is poured. It is performed for drying and permitting the escape of steam. Any die metal may be used, but the Doctor prefers Pastel's Babbit metal. The metal is poured in a semiplastic condition and tamped in the mold to avoid the spheroiding involved in the old procedure, when the metal had to be poured very hot. The nearer we come to the mouth the better will be the result. In the old process of sand molding there were several transfers, and each step involved changes and defects. In this process there are but two changes—the impression, and the pouring and pressing down of the moulten metal. Lead and tin are used for the counter die. The flasks are made in three parts. The impression is held in the lower part, filling in around it with the investment compound. The middle part of the flask is made to hold the metal. The impression is trimmed to relieve pressure in the proper places, as the metal model cannot be trimmed afterward. After drying and heating the investment portion, the Babbit metal is melted to mere fluidity, then stirred to make it plastic, poured into the impression and tamped down well to make it fill all portions well, and prevent spheroiding. It is cooled in water. Dry it well and smoke to prevent adhesion, and pour the counter die metal. There are four special advantages: (1) The short time required to make a die; (2) The low temperature at which the metal can be poured; (3) All irregularities can be taken sharply; (4) The ease of the process by which even a novice can get good results at once. —"Dominion Dental Journal."

---

DENTAL SINNERS.—One of the very many good clergymen,—"there are others"—once asked us "Why do you bother about quackery? Why don't you let these quacks alone?" to which we replied, "Why do you bother about sin? Why don't you let the sinners alone?" "But my mission is to save souls, yours only to save teeth," said he. "Very true," we remarked, "but do you think your moral code or religious creed will exempt from condemnation the dentist who deliberately lies, and who deceives and swindles people who entrust to him the care of their bodies? Is a thorough quack not as much a sinner against your belief as a common thief? There is, after all, some justification for the starving man who steals food, or the freezing man who steals fuel; but tell us, where do you find excuse for the dentist who deliberately wrongs and robs his patients? Now, if we succeed in making this dental sinner conscious of his iniquity, we make him a better subject for you; we do something towards helping you in the matter of his salvation. That seems to us logical. Isn't it theological?" Our clerical friend gave successive nods of apostolical approval so that question is settled.—"Dominion Dental Journal."

---

A NEW TEST FOR ALBUMEN.—Reagents for the detection of albumen in the urine must be colorless and must reveal the presence of albumen even when the amount is too small for quantitative estimation. Dr. Jolles has published a new and delicate test, consisting of:

| | |
|---|---|
| Chloride of mercury, - - | 10 grammes |
| Succinic Acid, - - - - | 20 " |
| Common Salt, - - - | 10 " |
| Distilled Water - - - | 500 " |

In the process of testing, 4 c. c. of the filtered urine are mixed with 1 c. c. of acetic acid, and 4 c. c. of the above reagent are added with shaking. In a second glass similar quantities of urine and acetic acid are mixed with pure water instead of with the reagent. This test yields a cloudiness of albumen in cases where the ordinary tests fail to give any result at all.—*The Lancet.*

---

LONGEVITY OF HUMAN LIFE INCREASING.—A German statistician has calculated that of every 1,000 persons 100 reach the age of 75, 38 the age of 85, and only two reach 95. In the seventeenth century the average duration of life was only 13 years; in the eighteenth, 20; in this century, 36.—*Medical News.*

LIQUID SILEX.—The solution known by this name, or a soluble glass, chemically the sodium silicate, ($Na_2 Si O_3$) is quite as effective a medium to prevent the adhesion of plaster to vulcanite as is tin foil, but certain precautions are necessary to procure the best results. The material should be kept in a moderately warm place and tightly stoppered. As soon as its viscidity becomes greater than a thin syrup, throw it away and buy a new bottle. Should it lose its perfect clearness discard it. The writer finds that about one-third of the four-ounce bottles in which it is sold is useful, the remainder is usually so deteriorated as to be worthless. Dilution with hot water and warming the solution restores its appearance, but for dental purposes, not its virtues. The model, after investment and also the teeth and entire investment, are freed of adherent wax by pouring over them a stream of boiling water. The excess of water is absorbed by means of bibulous paper. As soon as the wet appearance disappears from the plaster, it is ready to receive the silicate, not before.—"Ohio Dental Journal."

---

ATTACHMENT OF LOGAN CROWN.—Having prepared the root and obtained proper fit of crown to root end and correct articulation, smear the end of the root with a varnish of rosin and chloroform, and place in position a gutta-percha washer. Cover the crown with oxid of zinc in powder, warm it and force it into the gutta-percha, trimming off all excess. This secures perfect adaptation of crown to the root end.—P. W. Onderdonk, in *Southern Dental Journal.*

---

ITCHING IN ECZEMA.—Use a solution of permangnate of potassium in water, of 1 to 2 per cent., or possibly stronger in some cases. This is brushed or mopped over the surface and allowed to dry, which it does quickly. The pink-colored fluid turns soon to a dark-brown, and is finally thrown off by exfoliation of the tissues which it has oxidized.—L. D. Buckley, in *Medical Review.*

A NEW HEMOSTATIC.—Dr. Frohman, a dentist of Berlin, has recently given the profession the results of his experiments on ferripylin, and states that it has great hemostatic properties. Its application to more than 100 stubborn cases of bleeding gave excellent results. Post hemorrhage seldom resulted, and when it did further application caused permanent arrest. There are no painful after-effects, and it yields perfect coagulation. Ferripyrin is a combination of one part ferric chlorid, three parts antipyrin and five parts water. It is a dark red liquid, and can be purchased at the drug stores. It has an agreeable taste and is readily applied. In case of a lower extraction employ a small spoon and direct a sparing amount of the mixture into the socket, in the event of an upper case apply a saturated pellet of cotton to the socket. It gives immediate relief and one application usually suffices.—"Jour. fur Zahnheilkunde."

---

ICE TO RELIEVE DYSPEPSIA.—Pictet found that when dogs were plunged in a bath of low temperature, and kept there some time, they became ravenously hungry. Being himself a sufferer from stomach disease, he had forgotten what it was to have an appetite; so, wrapped in a thick pelisse, he descended tnto a refrigerating tank, the temperature being many degrees below zero. After four minutes he began to feel hungry; in eight minutes he climbed out of the tank with a painfully keen appetite. Many such experiments were made, and all the meals he took after a short stay in the refrigerator agreed with him. He found his dyspepsia was cured after the tenth descent.—*Ex.*

---

ANOTHER SOURCE OF INFECTION.—It is well known among oculists that the opera glasses, which may be hired in most theatres, frequently become the medium for spreading very serious eye diseases.—*Pop. Science News.*

